# Pediatric Dental Education Improves Interprofessional Healthcare Students’ Clinical Competence in Children’s Oral Health Assessment

**DOI:** 10.3390/dj7040106

**Published:** 2019-11-13

**Authors:** Remya Niranjan, JungSoo Kim, Brent Lin, Sheela Lewis, Punam Patel, Thuan Le, Abbey Alkon, Jyu-Lin Chen

**Affiliations:** 1 Division of Pediatric Dentistry, School of Dentistry, University of California, San Francisco, CA 94143, USA; remya.niranjan@ucsf.edu (R.N.); brent.lin@ucsf.edu (B.L.); sheela.lewis@ucsf.edu (S.L.); punam.patel2@ucsf.edu (P.P.); thuan.le@ucsf.edu (T.L.); 2 Department of Family Health Care Nursing, School of Nursing, University of California, San Francisco, CA 94143, USA; abbey.alkon@ucsf.edu (A.A.); jyu-lin.chen@ucsf.edu (J.-L.C.)

**Keywords:** pediatric dentistry, primary care, children’s oral health, interprofessional education, oral health education, public health dentistry, oral health disparity, access to care, clinical competency, oral health assessment

## Abstract

Primary care and healthcare providers can facilitate children’s timely referral to a dental home. However, there are few studies of providers’ oral health knowledge and clinical skills. This study aims to improve future healthcare providers’ knowledge, confidence, attitude, and clinical competence in assessing children’s oral health. Sixty-five health professional students participated in a 10-week didactic and clinical curriculum on children’s oral health. Fifty students completed pre- and post-training questionnaires and were assessed in their knowledge, confidence, and attitude. Calibrated examiners graded students’ clinical skills on a 24-point grading criterion. Descriptive statistics, paired sample *t*-test, and Pearson correlation were used in data analyses. Students were in dentistry (46%), nursing (28%), medicine (22%), and pharmacy (3%). Students significantly improved in knowledge (*t* = −7.71, *p* < 0.001), confidence (*t* = −10.30, *p* = <0.001), and attitude (*t* = −4.24, *p* = <0.001). Students on average scored 83% on clinical competence, with the highest average for fluoride varnish application (96%) and lowest for providing anticipatory guidance (69%). There was a moderate correlation between improvement in knowledge and their clinical skills (*r* = 0.39, *p* = 0.010). Interprofessional education improves students’ knowledge, confidence, attitude, and clinical competence in assessing children’s oral health. Such education is necessary in guiding future providers to gain adequate competence in serving children’s oral health needs.

## 1. Introduction

The American Academy of Pediatric Dentistry and American Academy of Pediatrics both recommend that infants be scheduled for an initial oral evaluation visit within six months of the eruption of the first primary tooth, but by no later than 12 months of age [1,2]. Despite recommendations, studies have shown that 90% of infants in the United States have seen a primary care provider, but only 2% have received an oral health evaluation before age 1 [3]. Furthermore, a study from 2008 demonstrated that children with public insurance coverage were 1.7 times more likely to have untreated dental caries than children not enrolled in state or government health insurance programs [4]. Data from the 1999–2004 National Health and Nutrition Examination Survey showed a prevalence of early childhood caries in 28% of children [5]. Moreover, 72% of tooth surfaces were untreated in 2–5 year-old children [5].

To improve access to oral health care and reduce oral health disparities in children, the American Academy of Pediatric Dentistry highly recommends the establishment of a dental home for children by 12 months of age [6]. Children with a dental home can receive appropriate preventive oral health care and can be screened for early and vital identification of oral disease. However, merely focusing on the establishment of a dental home as a viable measure to reduce caries has not been well supported with adequate evidence and may not be a feasible strategy [7]. Some potential barriers for the dental home strategy are lack of oral healthcare providers and dentists participating in the state welfare programs. In addition, very few general dentists are prepared and willing to treat infants and very young children [7,8]. Therefore, it is not enough to solely focus on a dental home model to combat access of care issues and oral health disparities in children.

Pediatric patients routinely see non-dental health care providers (such as pediatricians and pediatric nurse practitioners) earlier in life. This fact raises the importance of training primary health care providers in identifying oral health issues and making appropriate and timely referrals [9,10]. As such, incorporating oral health care into primary care trainings is ideal, and several schools, including the University of California, San Francisco, New York University, and University of Washington, have implemented interdisciplinary training programs [11,12,13]. However, with the recent innovation of these training programs, a review of literature shows few studies that validate clinical competency in oral health screenings and fluoride application. When 1407 medical multi-specialty physicians, residents, and nurses were surveyed, more than 80% answered knowledge-based questions correctly. However, less than 30% showed clinical competency for identifying tooth decay and oral pathology, and 95% reported having never applied fluoride varnish in their practice. Furthermore, 68% of medical providers reported making dental referrals “infrequently” [14].

With appropriate training, primary care providers could be effective partners in preventing and reducing oral health problems in children [15]. An interdisciplinary oral health education program has proven effective in training primary care students to adopt oral health assessments into their practice [16]. Post-training surveys show students improved significantly in their oral health knowledge, confidence in giving oral health counseling, and attitudes in including oral health examination into their practice. In their follow-up survey, 83% of students confirmed that they successfully incorporated oral health examinations into their well-child visits [17].

Numerous studies have shown successful incorporation of oral health training as part of interprofessional education; however, there is a lack of studies on the evaluation of clinical knowledge and the skills of these students [12,13,18]. The aims of this study are (1) to develop an interprofessional curriculum to improve knowledge, attitude, and confidence in providing children’s oral health care, (2) to assess students’ clinical competency in assessing children’s oral health, and (3) to evaluate whether improved knowledge is associated with actual clinical skills.

## 2. Materials and Methods 

This study has been approved by the University of California, San Francisco (UCSF) Committee on Human Research.

**Development of the Didactic and Clinical Curriculum:** A 10-week interprofessional pediatric oral health course for students in dentistry, nursing, medicine, and pharmacy was administered by an interdisciplinary faculty team. This course included weekly 1-h lectures for 10 weeks. Four lectures were delivered via pre-recorded online lectures, and 6 lectures (including case presentations and discussion session) were delivered in-class. The topics of these lectures included introduction on children’s oral health, oral health disparities, and clinical assessment and practice (Table 1). The students were required to attend a minimum of 1 clinical session (3.5 h per session) to observe a pediatric dentist, perform an oral health assessment of a child under the age of 14, and apply fluoride varnish under supervision of a faculty.

**Development of the questionnaire:** Questionnaires were developed to assess the change in students’ pediatric oral health knowledge, confidence, and attitude. Clinical skills assessment criteria were also developed to evaluate students’ clinical competence.

**(i) Demographics:** Students’ demographical information was collected, in addition to their current disciplines and year of study (Table 2).

**(ii) Knowledge:** The 11-item clinical knowledge questionnaire (Table 3) was developed and reviewed by the study team, including two pediatric dentists, one pediatric dental resident, and one pediatric nurse practitioner. One to two questions were designed to ask about the key objectives from each of the 10 lectures. The knowledge questionnaire was scored as correct (1) or incorrect (0), with 11 maximum points. Higher scores indicated better knowledge on children’s oral health.

**(iii) Confidence:** The 10-item confidence questionnaire (Table 3) was previously administered as part of an evaluation of an in-class oral health course for interdisciplinary students [17]. The questions assessed the students’ level of confidence in advising parents regarding different aspects of the child’s oral health. Students were given 3 answer choices, and each item was rated as 0 for not confident, 1 for somewhat confident, and 2 for very confident, with 20 maximum points. Greater points indicated greater confidence in advising parents on their child’s oral health.

**(iv) Attitude:** The 4-item attitude questionnaire (Table 3) [17]. The questions assessed students’ attitudes toward providing children’s oral health care. Students were given 4 answer choices (strongly disagree, disagree, agree, and strongly agree). Since most students answered either ‘agree’ or ‘strongly agree,’ answer choices ‘strongly disagree’ and ‘disagree’ were combined so that each item was rated as 0 for strongly disagree/disagree, 1 for agree, and 2 for strongly agree. Eight maximum points were available for the attitude questionnaire, with greater points indicating a more positive attitude towards providing children’s oral health care.

**(v) Clinical Competency:** Students were assessed on their clinical skills using the clinical skills assessment criteria (Table 4) that was developed for this evaluation project. Content and face validity were established by approved review of the items’ relevance to best practices and relevance to this oral health course by two pediatric dentists, one pediatric dental resident, and one pediatric nurse practitioner. To established inter-rater reliability, two examiners were asked to assess students’ performance based on the developed criteria. Two examiners separately assessed three students and reached 90% inter-rater reliability.

The clinical skills assessment criteria included 5 sections: assessment of the oral cavity (10 items), ability to identify caries and classify caries risk (3 items), application of topical fluoride varnish (4 items), providing age-appropriate anticipatory guidance (5 items), and providing appropriate follow-up care and referral to a dental home (2 items). Individual items were scored as ‘yes,’ ‘no’, or ‘not applicable.’ ‘Yes’ meant the student appropriately performed the task and was given a score of 1. ‘No’ meant the student did not perform the task and was given a score of 0. ‘Not applicable’ meant the student did not need to perform the task and explicitly indicated so to the examiner, therefore, was given the score of 1. For example, the student was given a point for N/A, if the student did not write a referral as the patient already had an established dental home. Twenty-four maximum points were available, with greater points indicating greater competence in clinical skills. Students were required to score at least 17 out of 24 points, as the minimum score to pass the course was 70%.

**Participants Recruitment and Questionnaire Administration:** Students were recruited from April 2018 to June 2019, with each quarter being 10 weeks long. Students were health professional students from the UCSF School of Dentistry, School of Nursing, School of Medicine, School of Pharmacy, and Touro University College of Osteopathic Medicine. All questionnaires were administered online via Qualtrics (Qualtrics, Provo, UT) before the first lecture (pre-test) and after the last lecture (post-test) to assess the change in students’ knowledge, confidence, and attitude [19]. Clinical sessions occurred half-way through the course, so that students could leverage the knowledge that they received from didactic lectures. A gold standard examiner observed and assessed students’ clinical skills using the assessment criteria.

**Statistical Analyses:** Data analyses were performed using SPSS 24.0 software (SPSS Inc., Armonk, NY, USA) [20]. Students’ demographics and characteristics were summarized with frequency and percentage. Total scores were computed for students’ knowledge, confidence, and attitude questionnaires for both pre- and post-test. A paired sample t-test was used to assess students’ pre- vs. post-training knowledge, confidence, and attitude scores. Students’ scores on the clinical skills assessment criteria were summarized with mean, standard deviation and percentage for each subsection and total score. Students’ improvement in knowledge, confidence, and attitude were computed by finding the difference between the pre- and post-training scores. Pearson correlation was used to assess correlation between students’ improvement in knowledge, confidence, and attitude vs. their clinical competence.

## 3. Results

### 3.1. Sample Characteristics

A total of 65 students were recruited to participate in this study (Table 2). The majority of participants were between 20 and 29 years old (68%), female (78.5%), Asian (59%), non-Hispanic or Latino (87%), had a family yearly income greater than $50,000 (44%) and a Bachelor’s degree (60%). Forty-one percent were first-generation college students, an underrepresented minority (22%), from a disadvantaged background (29%), and a rural residential background (14%). Sixty-five percent reported receiving a scholarship, financial aid (72%), and loans (55%). Students were an interprofessional group studying dentistry (46%), nursing (28%), medicine (21.5%), and pharmacy (3%). The majority were in the first year of their programs (48%).

### 3.2. Clinical Competency

According to the 24-item clinical skills assessment criteria, students showed the greatest competence during fluoride varnish application (96% correct for the subsection), caries risk assessment (90% correct), and assessment of oral cavity (85% correct). Students were the least competent in providing anticipatory guidance (69% correct) and devising a follow-up plan for the patient (77% correct). The mean total score for all sections was 83%. 

### 3.3. Knowledge, Confidence, and Attitude 

When compared pre- vs. post-test scores, students showed significant improvement in knowledge [mean (SD) = 6.57 (1.74) vs. 8.76 (1.33), *t* = −7.71, *p* < 0.001], confidence [mean (SD) = 8.00 (5.99) vs. 16.46 (3.33), *t* = −10.30, *p* = <0.001] and attitude [mean (SD) = 6.52 (1.79) vs. 7.52 (1.04), *t* = −4.24, *p* = <0.001] (Table 5).

### 3.4. Improvement in Knowledge, Confidence, and Attitude vs. Clinical Competence

A moderate correlation, but statistically significant, was found between students’ improvement in knowledge and their clinical skills assessment score [mean (SD) = 2.18 (1.94) vs. 20.02 (4.03), *r* = 0.39, *p* = 0.010] (Table 6). No significant correlation was found between students’ improvement in confidence and attitude vs. their clinical skills assessment score.

## 4. Discussion

This is one of the first studies that includes an objective, systematic approach in assessing future healthcare providers’ clinical competence while evaluating a pediatric oral health hybrid course. The evaluation shows a relationship between students’ improvement in knowledge and their actual clinical skills. This study found that interprofessional education significantly improved students’ knowledge, confidence and attitude in providing children’s oral health care. We also found that students acquired great competence in fluoride varnish application, caries risk assessment, and assessment of oral cavity, but not in providing anticipatory guidance and devising a follow-up plan. Improvement in knowledge was correlated with the student’s overall clinical competence. 

This study shows that students significantly improved in their knowledge, confidence, and attitude on children’s oral health after completion of the course. This is similar to previous studies where interprofessional education increased participants’ knowledge, confidence, and attitudes in children’s oral health [11,17]. This suggests that incorporating interdisciplinary training early on in the providers’ career can be a promising strategy in integrating children’s oral health care into primary care practices.

This study successfully assessed students with different professional training and their clinical competence in evaluating children’s oral health using a newly developed clinical skills assessment instrument. Among the competencies assessed, students showed the greatest competence in fluoride varnish applications. This is significant because fluoride varnish applications have been found efficacious in reducing incidence of early childhood caries [21] and can positively affect patient outcomes and reduce overall costs in a non-dental setting [7]. The U.S. Preventive Services Task Force has released a recommendation for primary care providers to apply fluoride varnish on all children starting at the age of primary tooth eruption [22]. However, despite the recommendation, only 4% of pediatricians regularly perform fluoride varnish applications. The lack of training was found to be the most common barrier in performing oral health-related activities [23]. The clinical curriculum implemented in the current study was successful in systematically training future healthcare providers to apply fluoride varnish as part of their routine oral health exams.

Students were also successful in assessing children’s oral cavities and determining their caries risk. This is consistent with the previous study that demonstrated how pediatric primary care providers, after two hours of training in infant oral health, were able to achieve adequate levels of accuracy in identifying cavitated carious teeth in children [24]. This is important because it was found that nearly 78% of primary care providers reported being most likely to make a dental referral for children who had signs of early decay or at high risk for future caries [25]. It is critical to train future healthcare providers in accurately assessing children’s oral health and determining the caries risk, as they will be more likely to refer such children to a dental home.

Students were the least competent in providing anticipatory guidance and formulating a follow-up plan for the patient (e.g., making a referral to a dental home). The curriculum has been revised to improve the clarity of the content. The American Academy of Pediatric Dentistry and the American Academy of Pediatrics recommend all children to establish a dental home by 12 months of age [6,26]. Despite the recommendations, there is a lack of adherence to the guidelines in reality. A study showed that pediatricians were able to identify 6.3% of children as high caries risk, but only 0.36 of them needing a dental referral [27]. Another study showed that 68% of medical providers reported ‘infrequently’ making dental referrals [14]. The possible rationales for low competence in providing anticipatory guidance and developing a follow-up plan may be due to the lack of sufficient contents in these areas in the curriculum. The curriculum has since been revised to address this deficiency. These findings suggest the need for further intervention in educating healthcare students to provide dental referrals as part of their routine practice.

A moderate correlation was found between students’ improvement in knowledge and their actual clinical skills. Other studies on interprofessional children’s oral health education also involved a combination of didactics and clinical simulations; however, limited studies measured participants’ clinical skills objectively on a set criterion [11,17,18,28]. No significant correlation was found between students’ improvement in confidence and attitude and their clinical skills. This finding is consistent with those of other investigators, and the lack of correlation between attitude, confidence, and competence requires further exploration [29,30,31]. Some possible explanations may include the validity of the competency assessment itself, the quality of the learning experience and learning environment, and the quality of feedback given during clinical experience leading up to the competency assessment [29].

This study has some limitations as a quasi-experimental study with no control group for comparison; therefore, no causation can be determined to see whether the students’ improvement in knowledge, confidence, and attitude is solely based on the implemented educational intervention. The sample size was also limited to 65 students, with only 50 students completing the course. Furthermore, there is no baseline measure of students’ clinical skills, as it is considered unethical to have students evaluate patients prior to training. Future studies should develop a methodology to measure level of participants’ baseline clinical skills and explore different means to improve participants’ clinical skills. In addition, future studies should compare clinical skills of students who have participated in interprofessional education to those who did not participate. These studies may also compare different healthcare settings and students’ disciplines.

This study is innovative because it evaluates students’ improvement in knowledge and its association with clinical skills level. To our knowledge, this is one of the first studies that systematically evaluated healthcare students’ clinical competence in evaluating children’s oral health. This curriculum can be replicated in various settings outside of school, including clinics in the community, to educate currently practicing healthcare providers. Such interprofessional education is necessary to guide future healthcare providers gain adequate knowledge, confidence, attitude, and clinical competence to serve children’s oral health needs.

## 5. Conclusions

Interprofessional children’s oral health education for healthcare students can improve their knowledge, confidence, and attitude. Furthermore, improvement in clinical knowledge is correlated with greater clinical skills in evaluating children’s oral health. Primary care providers are on the forefront of being able to help children establish a dental home because they are the first to see these young patients. Such education is necessary in guiding future providers to gain adequate clinical skills necessary to serve the broader population with children’s oral health needs.

## Figures and Tables

**Table 1:** Didactic lecture topics.

Week	Didactic Lecture Topics	Method	Duration
1	Introduction to children’s oral health and community dentistry	In-Class	1 h
2	Physical assessment of oral cavity and recognition of abnormalities	Online	1 h
3	Caries risk assessment and disease prevention	Online	1 h
4	Anticipatory guidance in pediatric dentistry	In-Class	1 h
5	Relationship between children’s oral health and overall systemic health	In-Class	1 h
6	Unconscious health bias and literacy	In-Class	1 h
7	Infant oral health care, dental home, and referral	Online	1 h
8	Oral health in special needs and vulnerable children	Online	1 h
9	Management of orofacial trauma and acute dental care	In-Class	1 h
10	Case presentations and discussion	In-Class	1 h

**Table 2:** Students’ demographics and characteristics.

Demographics and Characteristics	Frequency (%)
Age (in years):	
○ 20–29	44 (68%)
○ 30+	21 (32%)
Sex:	
○ Female	51 (78.5%)
○ Male	14 (21.5%)
Race:	
○ Asian	37 (59%)
○ White	16 (25%)
○ Other	10 (16%)
Ethnicity:	
○ Not Hispanic or Latino	54 (87%)
○ Hispanic or Latino	8 (13%)
Family yearly income:	
○ Less than $10,000	15 (25%)
○ Between $10,000–$49,000	19 (31%)
○ More than $50,000	27 (44%)
Highest education degree:	
○ Bachelor’s Degree	39 (60%)
○ Master’s Degree	15 (23%)
○ Other	11 (17%)
First-generation college student	26 (41%)
Underrepresented minority	14 (22%)
Disadvantaged background	18 (29%)
Rural residential background	9 (14%)
Are you receiving or have you ever received any of the following in the past?	
○ Scholarship	42 (65%)
○ Financial Aid	47 (72%)
○ Loan	36 (55%)
Currently enrolled educational program:	
○ Dental (Doctor of Dental Surgery (28), Master’s in Dental Hygiene (2))	30 (46%)
○ Nursing (Nurse Practitioner (12), Registered Nurse (4), Research Scholar (2))	18 (28%)
○ Medical (Doctor of Medicine (8), Doctor of Osteopathic Medicine (6))	14 (21.5%)
○ Pharmacy (Doctor of Pharmacy (2))	2 (3%)
○ Other (1)	1 (1.5%)
Year in enrolled program:	
○ 1st	31 (48%)
○ 2nd	15 (23%)
○ 3rd	14 (22%)
○ 4th and above	4 (6%)

**Table 3:** Knowledge, confidence, and attitude questionnaire and scoring.

**Knowledge Questionnaire (11 Points)**	**Answer Choice (Points)**
For a patient between 0–5 years with high risk for dental caries, how often can you apply fluoride varnish? Once a year Twice a year 3–4 times a year (Correct Answer) 6–12 times a year	Correct (1) Incorrect (0)
How much toothpaste should be applied on the toothbrush of a child between 3–6 years? Half-inch Pea-sized (Correct Answer) Three inches As much as necessary depending on the plaque	Correct (1) Incorrect (0)
At what age of the child does the parent NOT have to assist with tooth brushing? 2 years 4 years 6 years 8 years (Correct Answer)	Correct (1) Incorrect (0)
When do you advise the parent to start cleaning/brushing the child’s teeth at home? At birth By age 2 When first tooth erupts in the mouth (Correct Answer) When there are at least 5 teeth in the mouth	Correct (1) Incorrect (0)
A child with a white spot lesion in the mouth has a ___________ caries risk. Extreme High (Correct Answer) Moderate Low	Correct (1) Incorrect (0)
What is the most effective position for a provider to do a complete exam on a child under 1 year of age? Supine position on the table Knee-to-knee (Correct Answer) Patient sitting facing the provider on mother’s lap Infant lying on the examination table	Correct (1) Incorrect (0)
Which of the statements is true about xylitol? Pick 2 correct answers. □ Xylitol is a naturally occurring sugar that causes decay. □ Recommended dosage is 6–10 g/day. (Correct Answer) □ Xylitol is contraindicated in infants. □ Xylitol inhibits strep mutans in the mouth. (Correct Answer)	1 Correct Choice (0.5) 2 Correct Choices (1) Incorrect (0)
Bleeding from gums upon brushing is: Herpes Mucocele Gingivitis (Correct Answer) Strep throat	Correct (1) Incorrect (0)
At what age do you recommend fluoride supplements to prevent cavities? At birth 0–6 months 6 months-12 years (Correct Answer) 18–21 years	Correct (1) Incorrect (0)
The following are appropriate instructions to patients after application of fluoride varnish. Pick 2 answers that apply. □ Instruct patient not to drink hot liquids or eat hard foods. (Correct Answer) □ Instruct that patient might be adversely reacting to fluoride in case yellow or brownish staining occurs. □ Instruct patient not to brush/floss for at least 4–6 h (waiting until the next day is better). (Correct Answer) □ Instruct patient to remove the fluoride varnish with normal brushing and flossing at an appropriate time interval.	1 Correct Choice (0.5) 2 Correct Choices (1) Incorrect (0)
What is the correct sequence of applying fluoride varnish? Stir the varnish, paint the varnish, rinse the teeth, dry the teeth Stir the varnish, paint the varnish, dry the teeth, rinse the teeth Dry the teeth, stir the varnish, paint the varnish (Correct Answer) Rinse the teeth, stir the varnish, paint the varnish, dry the teeth	Correct (1) Incorrect (0)
**Confidence Questionnaire (20 points total)**	**Answer Choice (Points)**
How confident do you feel advising parents of infants and toddlers regarding: Their child’s oral hygiene Water fluoridation Dietary recommendations to prevent early childhood tooth decay Fluoride supplement during infancy/childhood Dental visits during infancy/childhood Examining teeth of infants and toddlers for tooth decay Identifying tooth decay in early childhood Identifying other signs of oral pathology Evaluating the risk of tooth decay in infants and toddlers Deciding if the child needs referral to a dentist	Very Confident (2) Somewhat Confident (1) Not Confident (0)
**Attitude Questionnaire (8 points)**	**Answer Choice (Points)**
Do you agree or disagree that the following should be part of routine well-child-care visits? Routine assessment for early signs of dental problems (e.g., dental decay, gingivitis) during the physical exam Referral to dentist by 1 year of age Counseling on the prevention of dental problems (e.g., dental decay, gingivitis, trauma) Prescription of fluoride supplements when indicated.	Strongly Agree (2) Agree (1) Disagree (0) Strongly Disagree (0)

**Table 4:** Clinical skills assessment criteria and scoring.

Clinical Skills Assessment Criteria (24 Points)	Answer Choice (Points)	Mean (SD) N = 50
A. Assessment of Oral Cavity (10 points) Proper positioning of the patient: Knee-to-Knee/Supine/Semi-supine/Upright Extraoral exam: Asymmetry Extraoral exam: Swelling Intraoral/soft tissue exam: Mucosa Intraoral/soft tissue exam: Tongue Intraoral/soft tissue exam: Lips Intraoral/soft tissue exam: Palate Oral hygiene: Plaque (heavy/moderate/low) Oral hygiene: Calculus (heavy/moderate/low) Gingiva (gingivitis)	Yes, student performed/identified correctly. (1) No, student failed to perform/identify. (0) N/A, student correctly mentioned non-applicable. (1)	8.52 (2.45)
B. Caries Risk Assessment (3 points) Visible caries identification: White spots Visible caries identification: Frank cavitation Caries risk: High/Moderate/Low		2.70 (0.68)
C. Topical Fluoride Application (4 points) Indication for fluoride varnish Fluoride application technique: Mucosa dried Fluoride application technique: Fluoride application technique Fluoride application technique: Post-op instructions		3.82 (0.56)
D. Anticipatory Guidance (5 points) Oral hygiene instructions Brushing/flossing technique Dietary counseling Non-nutritive sucking Injury prevention		3.44 (1.15)
E. Follow-Up Plan (2 points) Referral to dental home Recall periodicity		1.54 (0.73)
Total Score		20.02 (4.03)

**Table 5:** Pre- vs. post-test mean scores in knowledge, confidence and attitude.

Questionnaire	Pre-Test Mean (SD)	Post-Test Mean (SD)	*p*-Value *
Knowledge (N = 47)	6.57 (1.74)	8.76 (1.33)	<0.001 **
Confidence (N = 50)	8.00 (5.99)	16.46 (3.33)	<0.001 **
Attitude (N = 50)	6.52 (1.79)	7.52 (1.04)	<0.001 **

* Paired samples *t*-test. ** = statistically significant (*p* < 0.05).

**Table 6:** Correlation between improvement in knowledge, confidence, and attitude vs. clinical competence.

Questionnaire	Improvement Mean (SD)	Clinical Competence Mean (SD), N = 50	Pearson Correlation	*p*-Value *
Knowledge (N = 47)	2.18 (1.94)	20.02 (4.03)	0.386 (N = 44)	0.010 **
Confidence (N = 50)	8.46 (5.81)	20.02 (4.03)	0.258 (N = 48)	0.076
Attitude (N = 50)	1.00 (1.67)	20.02 (4.03)	0.183 (N = 48)	0.213

* Pearson correlation. ** = statistically significant (*p* < 0.05).
